# Low C3 Serum Levels Predict Severe Forms of STEC-HUS With Neurologic Involvement

**DOI:** 10.3389/fmed.2020.00357

**Published:** 2020-06-26

**Authors:** Giuseppe Stefano Netti, Luisa Santangelo, Leonardo Paulucci, Giovanni Piscopo, Diletta D. Torres, Vincenza Carbone, Paolo Giordano, Federica Spadaccino, Giuseppe Castellano, Giovanni Stallone, Loreto Gesualdo, Maria Chironna, Elena Ranieri, Mario Giordano

**Affiliations:** ^1^Unit of Clinical Pathology and Center for Molecular Medicine, Department of Medical and Surgical Sciences, University of Foggia, Foggia, Italy; ^2^Unit of Pediatric Nephrology, University Hospital Policlinico Consorziale - Giovanni XXIII, Bari, Italy; ^3^Post Graduated School in Pediatrics, University of Genoa, IRCCS Istituto Giannina Gaslini, Genoa, Italy; ^4^Nephrology Dialysis and Transplantation Unit, Department of Medical and Surgical Sciences, University of Foggia, Foggia, Italy; ^5^Nephrology Dialysis and Transplantation Unit, Department of Emergency and Organ Transplantation, University of Bari Aldo Moro, Bari, Italy; ^6^Department of Biomedical Sciences and Human Oncology, Hygiene Unit, University of Bari Aldo Moro, Bari, Italy

**Keywords:** hemolytic uremic syndrome, Shiga-toxin *E. coli*, neurologic involvement, complement system, C3 serum levels, Eculizumab

## Abstract

**Background:** The correlation between the severity of hemolytic uremic syndrome related to Shiga toxin–producing *Escherichia coli* (STEC-HUS) and involvement of the complement system has been examined in a small number of studies, with conflicting results. In the present study, we investigated whether serum C3 levels on admission are associated with neurologic involvement.

**Methods:** To this purpose, 68 consecutive STEC-HUS patients were recruited and main clinical and laboratory variables ad hospital admission were compared between those with or without neurologic involvement.

**Results:** STEC-HUS patients who developed neurologic involvement (NI) showed significant higher leukocyte count, C-reactive protein and hemoglobin, and lower sodium levels as compared with those without. Interestingly, baseline serum levels of C3 were significantly lower in patients with NI as compared with those without (*p* < 0.001). Moreover, when stratified according to need of Eculizumab rescue therapy due to severe NI, patients treated with this drug showed baseline C3 serum levels significantly lower than those who were not (*p* < 0.001).

Low C3 was independent risk factor for NI in our patients' population when entered as covariate in a multivariate logistic regression analysis including other major variables previously proposed as possible predictors of poor prognosis in STEC-HUS (for instance, leukocyte count, c-reactive protein, sodium levels) (HR 6.401, 95%CI 1.617–25.334, *p* = 0.008 for C3).

To underline the role of complement in the worsening of STEC-HUS patients' clinical conditions and outcomes, all patients were divided into two groups according to the baseline lower vs. normal serum levels of C3 and the main data on care needs were assessed. Interestingly more patients with lower C3 serum levels required renal replacement therapy (*p* = 0.024), anti-hypertensive therapy (*p* = 0.011), Intensive Care Unit admission (*p* = 0.009), and longer hospitalization (*p* = 0.003), thus displaying significantly more severe disease features as compared with those with normal C3 serum levels.

**Conclusions:** Our data suggests that children with STEC-HUS with decreased C3 concentrations at admission are more likely to develop neurologic involvement and are at increased risk of having severe clinical complications.

## Introduction

Hemolytic uremic syndrome (HUS) secondary to gastrointestinal infections due to Shiga toxin-producing *Escherichia coli* (STEC) is characterized by micro-angiopathic hemolytic anemia, thrombocytopenia, and renal injury (1). In children, STEC-HUS accounts one of the main causes of acute kidney injury (AKI); death occurs in 1–5% of affected patients while long-term renal sequelae are demonstrated in almost 30% of the survivors (2–4).

STEC-HUS mainly affects the kidney, but extra-renal complications are frequently described (5). The involvement of the central nervous system (CNS) often represents a life-threatening condition and it can result in severe long-term disability in HUS patients who overcome the acute phase of illness (6). For these reasons it's mandatory the early diagnosis of the STEC-HUS might require dedicated surveillance protocols (7); in addition, the discovery of early markers of disease severity is necessary in the attempt to promptly treat the patients and to reduce the risk of long term renal and extra-renal sequelae.

The endothelial damage caused by Shiga toxin (Stx) is more likely to be the culprit pathogenic mechanism of the disease (8); however, there is increasing evidence for complement system activation as a contributing factor involved in organ damage (9). Several reports during last decades have described low plasma C3 concentrations and augmented complement products' degradation in children affected by STEC-HUS (10–12). Recently an *in vitro* study showed that high doses of STX2 are able to induce direct activation of complement alternative pathway (AP) and to bind factor H, decreasing its activity on the cell surface (13). In addition, Morigi et al. demonstrated that alternative pathway activation of complement system by Stx promotes large C3a formation that triggers microvascular thrombosis (14). Moreover, complement activation was also inferred by the detection of circulating micro-vesicles derived from platelets, monocytes, and red blood cells bearing C3 and C9 in STEC-HUS patients (15, 16). More recently, deposits of C5b-9 were detected in renal tissues from STEC-HUS affected patients and additional studies revealed that Stx induces complement-mediated injury in glomerular endothelial cell and podocyte (17–19).

Despite all these relevant findings, only a few clinical studies have correlated the complement system activation with the clinical course. Furthermore, they presented conflicting results and most of them reported small series of patients, or even included patients without microbiological diagnosis (20–26).

Since 2011, we have incorporated serum C3 determination into the initial laboratory profile in patients with STECHUS; thus, we aimed to explore further the association between C3 concentrations on admission and severe neurologic involvement in a large cohort of patients with proven STEC infection.

## Subjects and Methods

### Patients

**Figure 1:**
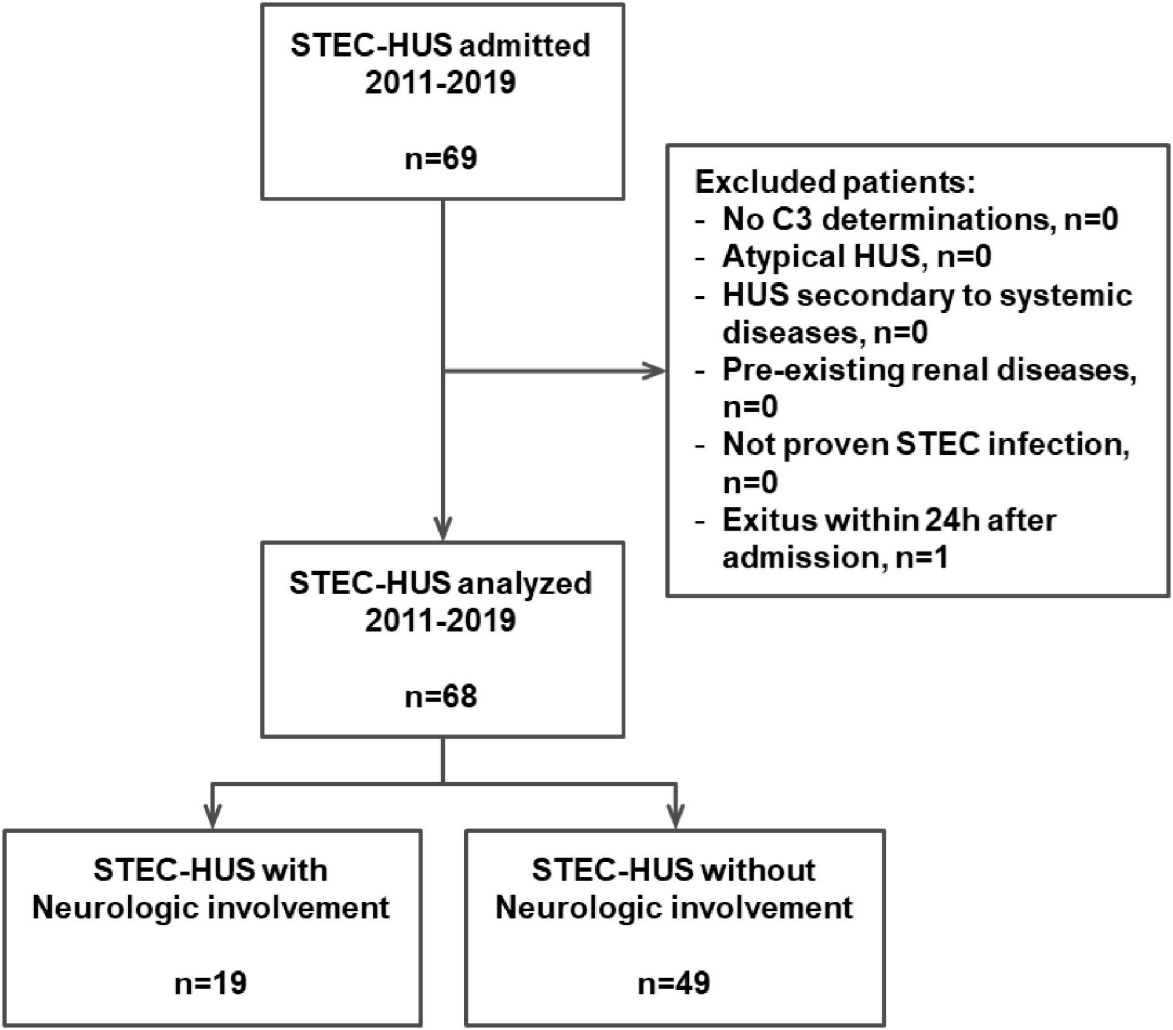
Algorithm of the study. STEC, Shiga toxin-producing *Escherichia coli*; HUS, hemolytic uremic syndrome.

In this retrospective single-center study, we included 69 consecutive children (33 males, 36 females) affected by STEC-HUS and treated at the Pediatric Nephrology and Dialysis Unit of the Pediatric Hospital “Giovanni XXIII” in Bari between January 2011 and December 2019. All the enrolled patients fulfilled the following criteria: (1) diagnosis of HUS with confirmed STEC infection; (2) age under 18 years old; and (3) C3 levels tested at admission. Patients with history of (1) recurrent or family history of hereditary HUS, (2) HUS associated with systemic diseases such as organ transplantation, systemic lupus erythematosus, pneumococcal infection, or HIV infection, and (3) pre-existing renal disease were excluded, as shown in Figure 1.

The main clinical and laboratory parameters were recorded at hospital admission and during the hospital stay in 68/69 children; an 18-month-old boy was excluded from further analysis due to death on the first day of hospitalization for severe gastrointestinal hemorrhage (Figure 1). Blood laboratory determinations performed and analyzed at admission were: white blood cell count (WBC), hemoglobin, hematocrit, platelets count, creatinine, c-reactive protein, sodium, potassium, lactic dehydrogenase (LDH), and albumin. Moreover, the C3 and the C4 concentrations were also assessed at hospital admission.

To assess the severity of the disease, the following data on care needs were collected: blood product administration, plasma-exchange, need for dialysis, treatment with antihypertensive drugs, intensive care unit admission, and days of hospitalization.

Among extra-renal complications, clinical manifestations of the central nervous system (CNS) involvement were recorded and assessed according to the Pediatric Neurologic Assessment Score for HUS (PNAS-HUS), previously described by Giordano et al. (27).

Patients with severe CNS involvement were treated with Eculizumab, after their parents signed informed consent for “off-label” use of this drug.

This retrospective single-center study was based on registry data. All procedures performed were in accordance with the 1964 Helsinki declaration and its later amendments. Ethical approval was obtained from the Institutional Review Board of the University Hospital “Policlinico Consorziale” of Bari (Italy) (Prot. 1624/2018). All the minors' legal guardians signed a written informed consent to collect their clinical data at time of hospital access and for the publication of any potentially identifiable images or data included in this article.

### Laboratory Methods

The diagnosis of STEC-HUS in children with clinical and laboratory signs of active thrombotic microangiopathy (low platelet count, hemolysis, and kidney damage) was confirmed by specific diagnostic assays: (a) Shiga toxin (Stx)-free fecal examination detection by the Vero cells assay; (b) isolation of Shiga toxin-producing *Escherichia coli* (STEC) with serological typing and PCR detection of the genes coding for virulence factors vtx1, vtx2, and eae; (c) specific anti-lipopolysaccharide serum antibodies (LPS) against the major STEC serotypes mainly related to typical HUS (O26, O157, O103, O111, and O145) (28, 29).

The C3 and the C4 concentrations were assessed by nephelometry (Image® 800 Beckman Coulter, Fullerton, CA, USA) and the normal reference values ranged within 0,79–1,52 g/L and 0,16–0,38 g/L, respectively.

### Statistical Analysis

Statistical analysis was performed using SPSS 25.0 software (SPSS Inc., Evanston, IL), as previously described (30–35). Normality of variable distribution was tested using Kolmogorov-Smirnov test. Comparison of variables between the different groups was obtained with Student's *t*-test and Mann-Whitney *U*-test owing to normal or non-parametric distribution. Frequencies were compared among groups by χ^2^-test. Correlation between two variables was ascertained by Pearson or Spearman's correlation tests, as appropriate.

A receiver operating characteristic (ROC) curve analysis was performed to validate the association of baseline C3 serum levels with neurologic involvement in STEC-HUS patients, and an operational cut-off level was defined to differentiate STEC-HUS patients at higher risk of CNS involvement. Subsequently all the patients were stratified according to baseline C3 cut-off level with the highest sensitivity and specificity for predicting neurologic involvement during the follow-up.

To test the independent effects of different variables on neurologic involvement onset, univariate and multivariate binary logistic regression analysis was used and partial correlation coefficients were computed and presented as hazard ratio and 95% confidence intervals (HR; 95%CI), as previously described (36, 37). Covariates included in the univariate binary logistic regression model were: age at admission, baseline leukocyte count, and baseline serum levels of C-reactive protein, hemoglobin, sodium, and C3. All the covariates were entered as quartiles, while C3 serum levels were entered as dichotomous variable. The variables were included in the multivariate analyses if they had a *p* < 0.05 in the univariate analysis or if they were clinically relevant confounders.

A *p* ≤ 0.05 was considered statistically significant. Results are expressed in the text as mean ± standard deviation, unless otherwise stated.

## Results

Between January 2011 and December 2019, 69 children (33 males, 36 females) (mean age 35 months, min 5 – max 197) were admitted to the Pediatric Nephrology and Dialysis Unit of the Pediatric Hospital “Giovanni XXIII” in Bari with a diagnosis of STEC-HUS. Due to a severe gastrointestinal hemorrhage, an 18-month-old boy died within the first day of hospitalization and was excluded from further analysis (Figure 1). All the 68 STEC-HUS patients fulfilled the inclusion criteria and were analyzed.

Median age of patients was 22 months and 33 (48.5%) were females. Time from first symptoms to STEC-HUS diagnosis was 5 days (2–9); bloody diarrhea was found in 86.8% of cases (*n* = 59). By stool culture and/or anti-lipopolysaccharide antibodies, serogroup O26 accounted for 38 cases, O111 for 13 cases, O145 for 9 case, O103 and O157 for 3 each. The remaining children were positive for O121 (2 cases) and O80 (only 1 case).

In 19/68 cases (27.9%), clinical manifestations of the central nervous system (CNS) involvement were recorded. Neurologic involvement (NI) was diagnosed along a median period of 5 days (minimum 2, maximum 10 days) after hospital admission when the following clinical manifestations were observed: alteration of consciousness, confusional state, disorders of communication skills, strabismus/eye fixing, nystagmus, amaurosis, seizures, hyporeactivity, disorders of muscle tone (hypo/hypertone), and neurovegetative system (heart rate, hypotension, respiratory rate alterations). The patients with NI was analyzed with clinical neurological evaluation, with electrophysiological investigations (EEGs) (19/19 cases), and when needed for severe neurologic sign, with neuroimaging techniques: in detail computer tomography of the brain was performed in 12/19 cases (63.2%), while magnetic resonance of the brain was performed in 15/19 cases (78.9%).

The mean age of children with neurologic involvement (9 males and 10 females) was 20 months, ranging between 8 and 109 months. All these patients were scored with PNAS-HUS assessment and their main clinical, laboratory, and therapeutic features are reported in Table 1.

**Table 1:** Main clinical, laboratory, and therapeutic information on STEC-HUS patients with neurologic involvement.

**Patient**	**Gender**	**Age**	***E. coli* strain**	**Days of hospitalization**	**Neurologic Involvement**						**Treatment**			**Outcome**
					Day of onset^*^	Clinical evaluation^**^	**EEG**	**NMR**	**CT scan**	**PNAS-HUS**	**Plasma-exchange**	**RRT**	**Eculizumab**	
1	M	25.1	O26	23	3	Yes	Yes	No	No	3,5	1	1	No	Discharge
2	M	20.4	O26	22	10	Yes	Yes	No	No	4	1	0	No	Discharge
3	M	15.5	O26	21	5	Yes	Yes	No	No	5	0	1	No	Discharge
4	M	18.3	O145	16	3	Yes	Yes	No	No	5	0	0	No	Discharge
5	F	17.0	O111	26	6	Yes	Yes	Yes	No	5,5	0	1	No	Discharge
6	F	26.6	O26	8	9	Yes	Yes	No	Yes	5,5	0	0	No	Discharge
7	F	53.3	O111	29	7	Yes	Yes	Yes	Yes	7,5	1	1	No	Discharge
8	M	13.0	O26	27	5	Yes	Yes	Yes	Yes	9	1	0	No	Discharge
9	M	19.8	O145	31	8	Yes	Yes	Yes	Yes	9	1	1	No	Discharge
10	F	22.8	O111	16	5	Yes	Yes	Yes	Yes	11,5	1	1	Yes	Discharge
11	M	7.7	O26	21	4	Yes	Yes	Yes	No	13	1	1	Yes	Discharge
12	F	17.1	O26	24	4	Yes	Yes	Yes	No	14	0	1	Yes	Discharge
13	F	19.5	O157	18	5	Yes	Yes	Yes	Yes	15	0	1	Yes	Discharge
14	F	16.1	O103	5	2	Yes	Yes	Yes	Yes	18	1	1	Yes	Death
15	M	45.7	O26	22	3	Yes	Yes	Yes	Yes	20	1	1	Yes	Discharge
16	F	109.2	O111	45	7	Yes	Yes	Yes	Yes	20	0	1	Yes	Discharge
17	F	19.8	O26	25	3	Yes	Yes	Yes	Yes	22	0	1	Yes	Discharge
18	F	24.7	O26	20	8	Yes	Yes	Yes	Yes	24	1	1	Yes	Discharge
19	M	33.0	O157	17	4	Yes	Yes	Yes	Yes	25,5	0	1	Yes	Discharge

* *Days of NI onset after hospital admission*.

** *Clinical manifestations observed at neurologic examination: alteration of consciousness, epileptic seizures, strabismus/eye fixing, nystagmus, virus disorders/amaurosis, disorders of muscle tone (hypo/hypertone), disorders of communication skills, neurovegetative system (heart rate, hypotension, respiratory rate alterations). CT, computed tomography; EEG, electroencephalogram; NMR, nuclear magnetic resonance; PNAS-HUS, Pediatric Neurologic Assessment Score for HUS; RRT, renal replacement therapy*.

Ten out of 19 showed severe neurologic involvement (PNAS-HUS >9) and, accordingly to previously proposed therapeutic recommendation (27), were effectively treated with Eculizumab, an anti-C5-convertase monoclonal antibody.

The main clinical and laboratory features of all patients after stratification in two groups according to presence or absence of CNS involvement are shown in Table 2.

**Table 2:** Clinical and laboratory characteristics of STEC-HUS patients with and without central nervous system (CNS) involvement.

**Variables**	**Total (*n* = 68)**	**CNS involvement (*n* = 19)**	**No CNS involvement (*n* = 49)**	P^*^
Age (months)	22 [5–196]	20 [8–109]	23 [5–196]	0.589
Gender (male/female), *n*	33/35	9/10	24/25	0.905
Blood pressure				
Systolic	105 [95–125]	105 [95–115]	108 [103–125]	0.773
Diastolic	65 [60–85]	70 [60–80]	65 [60–85]	0.863
Serum creatinine (mg/dL)	2.98 ± 2.42	2,76 ± 1.4	3.07 ± 2.71	0.637
Leukocyte count (×10^3^/μL)	16.9 ± 6.9	22.7 ± 8.0	14.6 ± 5.0	**<0.001**
C reactive protein (mg/dL)	30.9 ± 15.3	53.2 ± 17.8	22.3 ± 4.4	**0.021**
Hemoglobin (g/dL)	8.1 ± 1.2	8.7 ± 1.5	8.0 ± 1.0	**0.023**
Hematocrit (%)	27.0 ± 5.4	27.0 ± 4.4	27.0 ± 5.8	0.964
Platelets (×10^3^/μL)	51.2 ± 47.7	48.7 ± 37.5	52.1 ± 51.4	0.764
LDH (UI/mL)	3,068 ± 1,884	3,367 ± 2,076	2,952 ± 1,813	0.450
Sodium (mEq/L)	134.1 ± 5.4	131.1 ± 6.0	135.4 ± 4.6	**0.002**
Potassium (mEq/L)	4.3 ± 0.8	4.4 ± 0.9	4.3 ± 0.7	0.547
Albumin (g/dL)	3.1 ± 0.4	3.0 ± 0.3	3.1 ± 0.5	0.698
C3 (g/L)	0.83 ± 0.24	0.65 ± 0.19	0.90 ± 0.22	**<0.001**
C4 (g/L)	0.15 ± 0.07	0.13 ± 0.02	0.15 ± 0.06	0.313

* *CNS involvement vs. No CNS involvement. P-values in bold are statistically significant (p < 0.05)*.

In our study, at hospital admission STEC-HUS patients with neurologic involvement showed significantly higher leukocyte count (22.7 ± 8.0 vs. 14.6 ± 5.0 × 10^3^/μl, *p* < 0.001), C-reactive protein (53.2 ± 17.8 vs. 22.3 ± 4.4 mg/dL, *p* = 0.021), hemoglobin (8.7 ± 1.5 vs. 8.0 ± 1.0 g/dL, *p* = 0.023), and lower Sodium serum levels (131.1 ± 6.0 vs. 135.4 ± 4.6 mEq/L, *p* = 0.002), as compare with those without neurologic involvement (Table 2).

**Figure 2:**
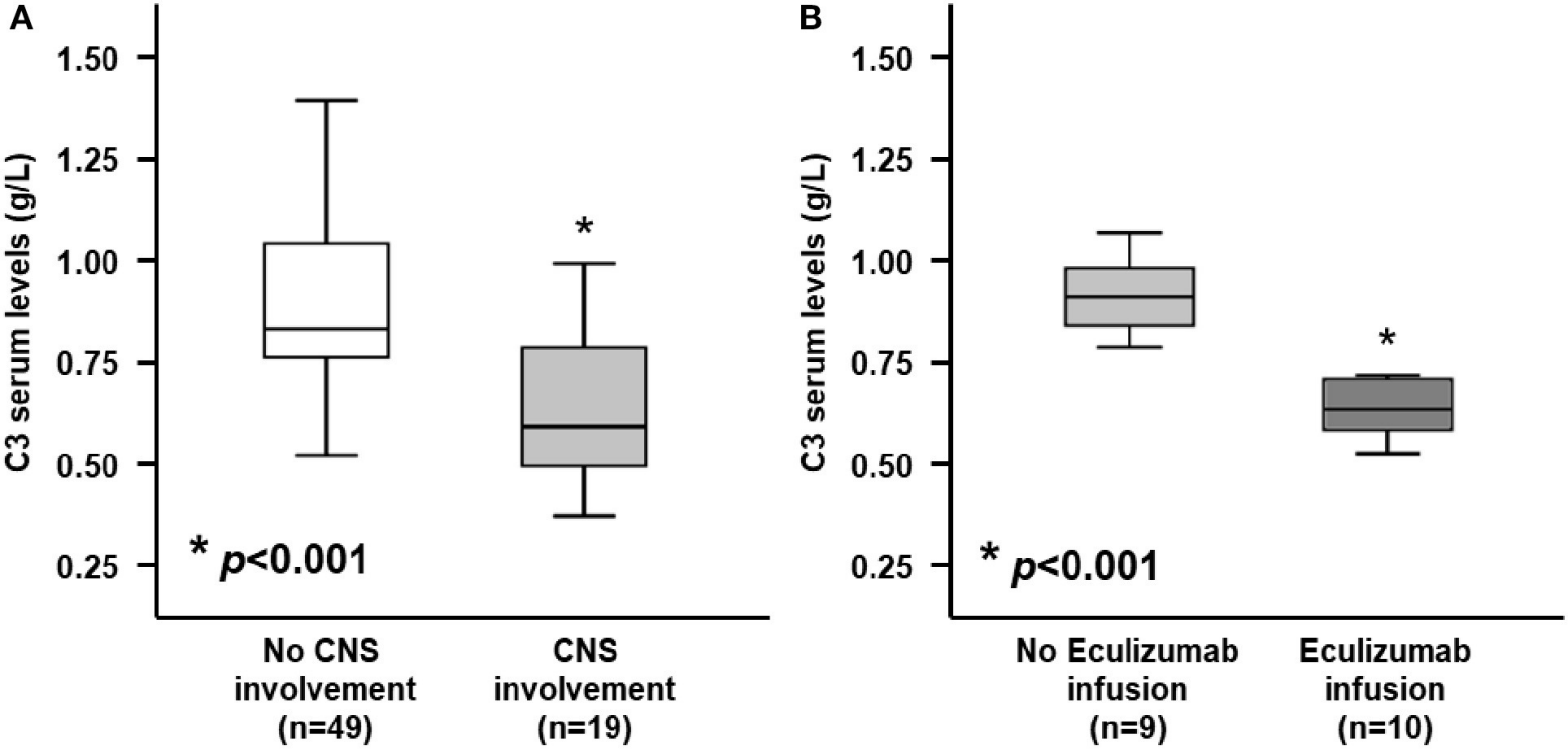
Serum C3 levels at admission between STEC-HUS patients depending on central nervous system (CNS) involvement and Eculizumab infusion. **(A)** At hospital admission, patients who would develop neurologic involvement (light gray box), showed significantly lower serum C3 levels, as compared with those who would not develop neurologic involvement (white box) (0.65 ± 0.19 vs. 0.90 ± 0.22 g/L; *p* < 0.001). **(B)** Among patients who would develop neurologic involvement, those who needed rescue therapy with Eculizumab (dark gray box) showed significantly lower C3 levels, as compared with those who did not need it (light gray box) (0.50 ± 0.08 vs. 0.82 ± 0.11 g/L; *p* < 0.001). Mann-Whitney *U*-test for non-parametric data. Data in the graphs are expressed as median and 25th and 75th percentiles in boxes and 5th and 95th percentiles as whiskers. *p < 0.001.

Interestingly baseline serum levels of C3 fraction, but not C4, were significantly lower in patients with neurologic involvement as compared with those without neurologic involvement (0.65 ± 0.19 vs. 0.90 ± 0.22 g/dL, *p* < 0.001; 0.13 ± 0.02 vs. 0.15 ± 0.06 g/dL, *p* = 0.313, for C3 and C4, respectively; Table 2 and Figure 2A). Moreover, when stratified according to need of Eculizumab rescue therapy due to severe neurologic involvement, patients treated with this drug showed baseline C3 serum levels significantly lower than those who has not be treated (0.50 ± 0.08 vs. 0.82 ± 0.11 g/L, *p* < 0.001; Figure 2B). Of note the severity of neurologic involvement in patients with lower C3 levels was underlined by negative correlation between this parameter and the PNAS-HUS score (Pearson's correlation coefficient = 0.993, *r*^2^ = −0.9181, *p* < 0.001).

**Figure 3:**
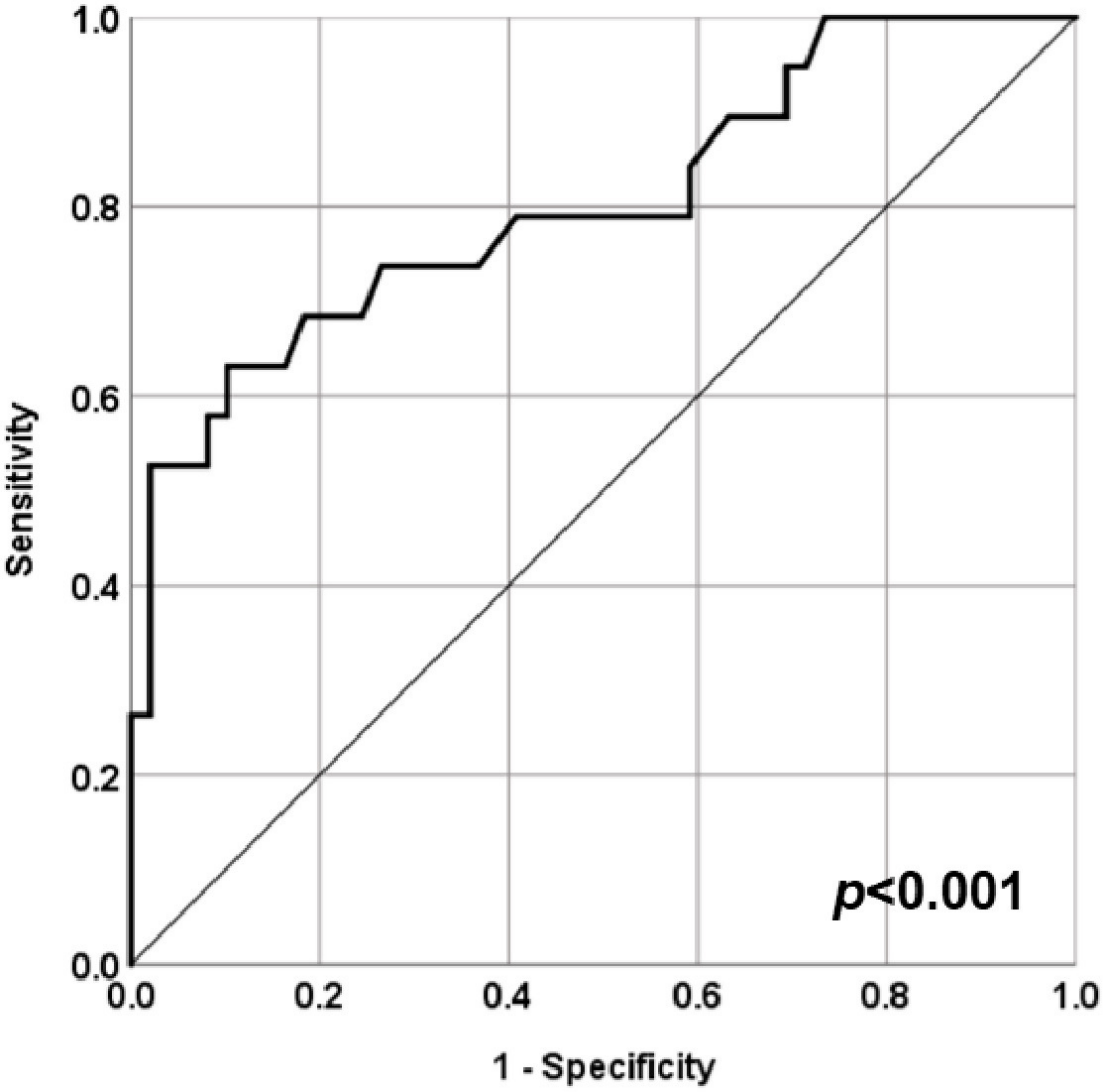
ROC curve for serum C3 levels at admission and central nervous system (CNS) involvement in STEC-HUS patients. ROC curve analysis to evaluate the predictive role of baseline serum C3 levels as risk factor for neurologic involvement in STEC-HUS patients (AUC = 0.804, CI 95% 0.678–0.930, *p* < 0.001).

A ROC curve analysis was performed to evaluate the predictive role of baseline serum C3 levels as risk factor for neurologic involvement in STEC-HUS patients. The analysis showed that a C3 serum level of 0.765 g/dL as the best cut-off value associated with the higher risk to develop CNS involvement in STEC-HUS patients with an 73.1% specificity and a 73.5% sensitivity (AUC = 0.804, CI 95% 0.678–0.930, *p* < 0.001; Figure 3).

To estimate the relative risk for central nervous system (CNS) involvement in STEC-HUS patients showing serum C3 level above or below the cut-off value (0.765 g/L), a binary logistic regression analysis was performed using onset of neurologic involvement as dependent variable, and patient's age, leukocyte count, C-reactive protein, hemoglobin, sodium, and C3 serum levels at baseline as covariates (Table 3).

**Table 3:** Univariate and multivariate binary logistic regression for central nervous system (CNS) involvement in STEC-HUS patients.

**Variables**	**Category**	**Univariate analysis**				**Multivariate analysis**			
		**HR**	**CI 95%**		***P***	**HR**	**CI 95%**		***P***
			**Lower**	**Higher**			**Lower**	**Higher**	
Age	Quartiles	0.864	0.536	1.009	0.546	NA	NA	NA	NA
Leukocyte count	Quartiles	2.719	1.478	5.000	**0.001**	2.355	1.136	4.882	**0.021**
C-reactive protein	Quartiles	1.686	1.011	2.811	**0.045**	1.108	0.536	2.293	0.782
Hemoglobin	Quartiles	1.388	0.852	2.263	0.188	NA	NA	NA	NA
Hyponatremia	Quartiles	2.227	1.276	3.888	**0.005**	2.049	1.071	3.918	**0.030**
C3 serum levels	<0.765 vs. >0.765 g/L	7.75	2.331	25.796	**0.001**	6.401	1.617	25.334	**0.008**

*CI, confidence interval; HR, hazard ratio. P-values in bold are statistically significant (p < 0.05)*.

Univariate analysis showed that the following covariates affected neurologic involvement onset in STEC-HUS: Leukocyte count (HR 2.719, 95%CI 1.478–5.000, *p* < 0.001), C-reactive protein (HR 1.686, 95%CI 1.011–2.811, *p* = 0.045), Hyponatremia (HR 2.227, 95% 1.276–3.888, *p* = 0.005) and C3 serum levels (HR 7.75, 95%CI 2.331–25.796, *p* < 0.001). As shown in Table 3, the results of the multivariate analysis confirmed a significant effect on neurologic involvement onset all of C3 levels, Leukocyte count and Hyponatremia at baseline, while the other were not significant (HR 6.401, 95%CI 1.617–25.334, *p* = 0.008 for C3; HR 2.355, 95%CI 1.136–4.882, *p* = 0.021 for Leukocyte count; HR 2.049, 95%CI 1.071–3.918, *p* = 0.030 for Hyponatremia).

Finally, to further evaluate the influence of complement activation on STEC-HUS patient's clinical conditions and outcomes, all patients were assigned to two groups according to the baseline serum levels of C3 above or below the cut-off value found in the whole study group (0.765 g/L). Interestingly patients with lower C3 serum levels required more frequently renal replacement therapy (74.1 vs. 46.3%, *p* = 0.024), anti-hypertensive therapy (51.8 vs. 21.9%, *p* = 0.011), Intensive Care Unit admission (22.2 vs. 2.4%, *p* = 0.009), and longer hospitalization (19.8 ± 8.1 vs. 14.8 ± 5.1 days, *p* = 0.003), as compared with patients with normal C3 serum levels (Table 4). Noteworthy, baseline serum levels of C3 in STEC-HUS patient negatively correlated with days of hospital stay (Pearson's correlation coefficient = 0.373, *r*^2^ = −0.1426, *p* = 0.002).

**Table 4:** Clinical and laboratory characteristics of STEC-HUS patients with low vs. normal C3 serum levels at baseline.

**Variables**	**Total (*n* = 68)**	**C3 <0.765 g/L (*n* = 27)**	**C3 >0.765 g/L (*n* = 41)**	P*
RBC/PLT infusion (yes/no)	66/2	27/0	39/2	0.244
Plasma-infusion (yes/no)	59/9	25/2	34/7	0.250
Plasma-exchange (yes/no)	18/50	9/18	9/32	0.298
Renal replacement therapy (yes/no)	39/29	20/7	19/22	**0.024**
Anti-hypertensive drugs (yes/no)	23/45	14/13	9/32	**0.011**
Intensive Care Unit admission (yes/no)	7/61	6/21	1/40	**0.009**
Days of Hospital stay (*n*)	16.8 ± 6.8	19.8 ± 8.1	14.8 ± 5.1	**0.003**

*P-values in bold are statistically significant (p < 0.05)*.

## Discussion

The results of the present study suggest that decreased serum C3 concentrations, assessed at hospital admission, were significantly associated with CNS involvement in STEC-HUS. The patients, who would develop neurologic involvement, at hospital admission showed significant alterations of specific laboratory parameters (higher leukocyte count, higher c-reactive protein, higher hemoglobin, lower sodium) which had been related to poor prognosis or more severe disease (38). Among them, serum levels of C3 were significantly lower in patients with CNS involvement, as compared with those without neurologic signs. More interestingly, patients with neurologic involvement, who required rescue therapy with Eculizumab (an anti-C5-convertase monoclonal antibody), showed even lower levels of C3, as compared with patients with mild neurologic impairment which were not treated with this drug.

Despite the role of complement in STEC-HUS physiopathology had been widely analyzed in recent years, clinical data supporting a key role of complement activation affecting the course of illness are still limited. Moreover, the conclusions of many pediatric studies linking complement activation and disease evolution are conflicting. While some studies showed a relationship between increased activity of complement alternative pathway and poor prognosis, other reports denied such association or just observed trends toward more severe disease, which did not reach statistically significant differences probably due to the small sample size analyzed (20–26).

In our study, low levels of C3 seemed to be significantly related not only with CNS involvement *per se*, but also with severe forms of neurologic involvement. To confirm this potential predictive role, lower baseline C3 serum levels were inversely correlated with the severity of neurological observation, as assessed by PNAS-HUS score (27).

Noteworthy, low C3 was an independent risk factor for neurologic involvement in our patients' population even as entered as covariate in a multivariate logistic regression analysis including other major variables previously proposed as possible predictors of poor prognosis in STEC-HUS (for instance, leukocyte count, c-reactive protein, sodium levels).

In this model, only leukocytosis and hyponatremia also resulted independently correlated with higher risk of CNS manifestations in these patients. Previous studies had shown that leukocytosis was associated with a higher risk of poor prognosis (39, 40) and hyponatremia was even identified as a predictor of death (41). Probably our findings underline that clinical neurologic outcome depends not only by the direct effects of Stx but also by secondary effects due to activation of the complement system (9, 23) and by neuronal cells dehydration due to hyponatremia (41, 42).

In univariate analysis, patients with higher hemoglobin concentration and C-reactive protein were at higher risk of neurologic involvement. These parameters are, respectively, an acute phase protein and a surrogate marker of dehydration and have been repeatedly associated with adverse outcomes in STEC-HUS patients (43–45). In our study, however, these laboratory markers did not significantly affect the development of CNS involvement in the multivariate model, probably due to the key role of alternative pathway activation in the onset of thrombotic microangiopathy onto brain microvascular endothelial cells (46, 47).

An intriguing issue to be addressed includes the different decrease in complement factors C3 and C4 serum levels. In our study baseline serum levels of C3 fraction, but not C4, were significantly lower in patients with neurologic involvement. Although decreased C4 levels have been occasionally reported in some patients (23, 48), its significance remains unclear probably due to the lack of evidence in the activation of classical and/or lectin pathways in STEC-HUS (9, 14, 22, 49).

To underline the possible role of complement C3 levels in the worsening of STEC-HUS patient's clinical conditions and outcomes, all patients were divided into two groups according to the baseline serum levels of C3 above or below the cut-off (0.765 g/L) and the main data of clinical and therapeutic management were evaluated. Interestingly patients with lower C3 serum levels required more frequently renal replacement therapy, anti-hypertensive therapy, Intensive Care Unit admission and longer hospitalization, thus displaying significantly more severe disease phenotypes as compared with those with normal C3 serum levels. These observations are in line with multiple previous reports which underline the key role of alternative pathway activation in the onset of organ damage during STEC-HUS (14, 50).

Taken together, these results suggest the potential role of low C3 serum level as reliable marker of both neurologic involvement onset and diseases severity in STEC-HUS patients.

Although this study encompasses a larger series of children with microbiologically diagnosed STEC-HUS and analyzes the association between C3 concentrations and disease outcomes, some limitations deserve consideration.

The monocentric retrospective analysis and the rather limited number of cases might limit our observations. However, further prospective multicenter studies are warranted to confirm our findings. Moreover, we measured C3 level, but not complement pathway fragments (i.e., soluble C5b9). Furthermore, C3 titration during the follow-up was not performed. However, our data show that C3 determination is a readily available assay in most laboratories and able to predict neurologic involvement onset and diseases severity in STEC-HUS patients.

## Conclusions

Our data suggests that children with STEC-HUS with decreased C3 concentrations at admission are more likely to develop neurologic involvement and are at increased risk of having severe clinical complications.

## Data Availability Statement

The raw data supporting the conclusions of this article will be made available by the authors, without undue reservation.

## Ethics Statement

Ethical approval was obtained from the Institutional Review Board of the University Hospital Policlinico Consorziale of Bari (Italy) (prot. 1624/2018). Written informed consent to participate in this study was provided by the participants' legal guardian/next of kin.

## Author Contributions

GN and LS conceived and designed the study, analyzed the data, and drafted the manuscript. LP, GP, DT, VC, and PG collected the clinical data and helped to interpret the results. FS and GC analyzed the data, interpreted results, and prepared the figures. GS, LG, MC, and ER helped to draft the manuscript. MG edited and revised manuscript and approved the final version of manuscript. All authors contributed to the article and approved the submitted version.

## Conflict of Interest

The authors declare that the research was conducted in the absence of any commercial or financial relationships that could be construed as a potential conflict of interest.
